# Factors associated with sleep quality among nurse managers during regular prevention and control of the COVID-19 in China: a cross-sectional study

**DOI:** 10.1186/s12912-022-01149-w

**Published:** 2022-12-19

**Authors:** Rong Chen, Pei Fang, Lanhui Tan, Jia Li, Liping Yu

**Affiliations:** 1 Department of Pediatric Intensive Care Unit, Xiangyang Central Hospital, Affiliated Hospital of Hubei University of Arts and Science, No.136 Jingzhou Street, Xiangcheng, Xiangyang, 441021 Hubei China; 2 School of Nursing, Wuhan University, No.115 Donghu Road, Wuchang, Wuhan, 430071 Hubei China

**Keywords:** Nurse managers, Sleep quality, Sleep disorders, Coping style, COVID-19, Regular epidemic prevention and control

## Abstract

**Background:**

Nurse managers play a pivotal role in quality patient care and staff satisfaction and retention. An overwhelming amount of work tasks and responsibilities might result in their sleep problems which are expected to aggravate in the context of the COVID-19, thereby affecting their overall health and work quality. However, little attention has been paid to sleep quality among nurse managers. This study aimed to investigate the prevalence of sleep disorders among nurse managers and identify related factors of sleep quality during regular prevention and control of the COVID-19 pandemic in China.

**Methods:**

This cross-sectional online survey was conducted in 14 hospitals on a sample of 327 nurse managers in China. Participants were invited to complete the general demographic questionnaire, Pittsburgh Sleep Quality Index (PSQI) and Simplified Coping Style Questionnaire (SCSQ). Multiple linear regression analysis was used to explore the influencing factors of sleep quality among nurse managers.

**Results:**

In this study, 43.7% of nurse managers suffered from sleep disorders. Active coping style and frequent exercise were positive factors that could influence nurse managers’ sleep quality, while passive coping style and age over 41 years old were negative predictors, collectively accounting for 52.0% of the variance of sleep quality.

**Conclusions:**

The issue of sleep disorders among nurse managers during regular epidemic prevention and control period is underscored. Coping style and demographic factors including age and frequency of exercise can significantly affect nurse managers’ sleep quality. Healthcare administrators should pay more attention to nurse managers’ sleep disorders and implement targeted strategies based on influencing factors to ensure their sleep quality.

## Background

The novel coronavirus disease 2019 (COVID-19) spread rapidly throughout China and elsewhere, becoming a major public health event [1]. COVID-19 outbreak has constituted a massive challenge to already strained health systems worldwide [2]. Hospitals have remained on the frontline of prevention and control since the COVID-19 outbreak, and all medical staff have been under great pressure [3]. Since April 2020, the pandemic in China has been well controlled, and China has moved into the stage of regular pandemic prevention and control [4]. Even so, there are still new sporadic instances of local and imported cases in China, and the overall situation of the current global pandemic is still severe, which places demands on nurse managers.

As one of the most important administrative members of the healthcare team, nurse managers must allocate scarce resources effectively, create supportive work environments for nurses, and deal with rapidly changing guidelines related to COVID-19 [5]. In addition, they have 24-h responsibilities for their units every day and are always on-call [6, 7]. A heavy workload and complex duties expose nurse managers to huge stress and can further lead to the sleep disorders, such as insomnia, somnolence and short sleep duration [8]. Sleep disorders may result in serious health consequences of nurse managers and further the poor work performance and unsatisfactory decision-making [9], while it may have been a neglected issue.

Before the COVID-19 outbreak, several studies explored nurse managers’ sleep problems. For example, Sigursteinsdóttir et al. (2020) conducted an online survey in Iceland and found that 44% of 110 nurse managers experienced short sleep duration and 12% of them rarely had adequate sleep [10]. A study with 126 Chinese nurse managers found that the prevalence of poor sleep quality was 42.9% [11]. In addition, a qualitative study in America also showed that nurse managers reported sleep problems generally [7]. However, there is a paucity of research on nurse managers’ sleep quality in the context of COVID-19. In China, regular epidemic prevention and control has added much management stress to nurse managers, such as organizing nucleic acid testing for personnel and patients regularly, performing regular training, inspection and assessment related to the epidemic, and implementing strict channel management in the ward [3]. Thus, the sleep disorders of nurse managers during this period are expected to be exacerbated, compared to normal times. It has been reported that sleep disorders were associated with considerable diseases and functional impairment [7]. For nurse managers, sleep disorders were associated with chronic fatigue, anxiety and depression, and in the long term, they can adversely affect their quality of life, work efficiency and job satisfaction [12–14]. Therefore, it is extremely important to evaluate, prevent and address nurse managers’ sleep disorders to promote their physical and mental health and ensure the quality of work.

Previous studies have shown that personal factors (e.g., age, gender, marital status and lifestyle), environmental conditions, psychological diseases and other factors can influence individuals’ sleep quality [15, 16]. In recent years, there is increasing evidence that coping style might be an independent and important predictor of sleep quality [17–20]. Coping refers to stable cognitive and behavioral strategies adopted by individuals in response to external and internal challenges or stressors, which can be divided into two styles: active coping and passive coping [21]. Active coping style is manifested by the adaptive responses, such as considering ways to solve problems, express emotions to friends or families and seek support from others, while passive coping style refers to dealing with stress and problems by neglecting, avoidance and denial [22]. The COVID-19 pandemic has put additional tremendous stress and challenges on nurse managers who have been inevitably accompanied by the process of coping. However, the studies that explored the effect of coping style on sleep quality are still limited and there is a lack of research on this relationship among nurse managers. Therefore, understanding the relationship between coping style and sleep quality in nurse managers may provide the foundation for the formulation of strategies to improve their sleep quality.

This study was designed to investigate the prevalence of sleep disorders and identify factors predicting sleep quality among nurse managers in China during regular prevention and control of the COVID-19.

## Methods

### Study design, setting and sample

A cross-sectional online survey was used to collect data from nurse managers. It was conducted in 14 public COVID-19 designated hospitals (6 tertiary hospitals and 8 secondary hospitals) from May 10–13 2020 in Xiangyang City, Hubei Province, China. During data collection, there were no coronavirus cases in most cities in China, including Xiangyang. Participants were selected adopting the convenience sampling technique. The sample size was calculated according to the formula *N* = *Z*^2^_1-α/2_ × σ^2^ / δ^2^, where *N* = initial estimated sample size, *Z* = confidence level (α) [23]. We assumed the type I error (α) of 5% usually, and both standard deviation (σ) and precision level (δ) were determined as 3 and 0.35 points respectively based on previous studies [11, 13]. Then the minimum sample size required was 283 (1.96^2^ × 3^2^ / 0.35^2^). Finally, the total sample size was increased to 312 considering a likely attrition rate of 10%. The inclusion criteria of nurse managers were as follows: (a) having registered nurse licenses; (b) working as a nurse manager and employed full-time; (c) having at least one year of work experience as a nurse manager. The nurse managers who were on vacation or had severe physical or mental problems were excluded.

### Instruments

#### General demographic questionnaire

The self-designed general demographic questionnaire includes gender, age, marital status, educational level, professional title, seniority in a nurse manager position, frequency of exercise and whether to keep a 24-h mobile phone on.

#### Pittsburgh sleep quality index (PSQI)

The sleep quality of respondents was assessed by the Pittsburgh Sleep Quality Index (PSQI) [24]. This scale was translated into Chinese and revised by Liu et al. (1996) [25], which has been widely used across the Chinese population. It contains 19 items, consisting of 7 dimensions: subjective sleep quality, sleep latency, sleep duration, sleep efficiency, sleep disturbance, sleep medication use and daytime dysfunction [24]. Each item is assessed on a 4-point Likert scale and the total score ranges from 0 to 21, with a higher score indicating a higher level of sleep disorders or worse sleep quality. Previous studies often used 5 as the cut-off value for sleep disorders, while recent studies suggested that the cut-off value of 7 showed higher sensitivity and specificity in identifying sleep disorders in the Chinese population [8, 26, 27]. Therefore, a value > 7 of the PSQI score was considered as the presence of sleep disorders in our study. The Chinese version of PSQI was reported good internal consistency with a Cronbach’s alpha coefficient of 0.842 by Liu et al. (1996) [25].

#### Simplified coping style questionnaire (SCSQ)

Nurse managers’ coping style was measured by the Simplified Coping Style Questionnaire (SCSQ). The SCSQ, based on the Ways of Coping Questionnaire designed by Folkman & Lazarus [28], was developed and revised into Chinese by Xie (1998) [29]. It consists of 20 items covering two dimensions (active coping and passive coping), and each item is rated on a 4-point Likert scale from 0 (“never”) to 3 (“always”). There are 12 and 8 items incorporated in active coping and passive coping dimensions respectively. Higher scores represent higher frequencies of relevant coping styles. The total scale of SCSQ and active and passive coping subscales were all shown to be valid and reliable in Xie (1998)’s study [29], with the Cronbach’s alpha coefficients of 0.90, 0.89 and 0.78 respectively.

### Data collection

Data were collected from May 10–13 2020. The questionnaire survey was conducted by Wen Juan Xing (www.wjx.cn), an online data collection platform. To ensure the accuracy of the data, all items were set as required questions to ensure the completeness of the questionnaires, and each IP address or device was limited to submit the questionnaires only once for avoiding repetition. Researchers firstly contacted the directors of nursing departments of 14 hospitals before data collecting, explicated the objectives and some details of our study and invited them to advertise this study among nurse managers employed in their hospitals. Then we sent the link of the online anonymous questionnaires to the director of each hospital’s nursing department through We Chat (a popular social application with over 1 billion active users in China). And directors of nursing departments distributed the link respectively to their private We Chat groups comprised of nurse managers. An explanatory statement, outlined at the head of the questionnaires, was provided for the informed consent of participants. It took approximately 15–20 min for the participants to complete the questionnaires. Data from Wen Juan Xing were imported into Excel for screening. A total of 334 nurse managers completed the questionnaires. After excluding questionnaires that took less than 5 min to complete (*n* = 4) and the answers were illogical or contradictory (*n* = 3), 327 nurse managers were included for analysis, with an effective response rate of 97.90%.

### Data analysis

The IBM SPSS Statistics 25.0 was used for statistical analysis. The categorical variables of the demographic characteristics were described by frequency and percentage, while continuous variables were presented as mean and standard deviation. The independent sample t-test and one-way analysis of variance (ANOVA) were used to compare the differences in sleep quality between nurse managers with different demographic characteristics. The Pearson’s correlation coefficient was computed to examine the association between sleep quality and coping style. And multiple linear regression analysis was applied to identify the influencing factors of sleep quality. All statistical tests were two-sided, with a *p-*value less than 0.05 considered statistically significant.

### Ethical considerations

This study was approved by the Ethics Committee of Xiangyang Central Hospital (NO. 2020–031). After reading the objectives and information of the survey presented at the head of the online questionnaires, nurse managers could voluntarily continue or withdraw from filling out them. Nurse managers who completed and submitted the online questionnaires were considered to have provided informed consent to participate in this study. All data collected from participants were anonymous and held confidentially.

## Results

### Demographic characteristics

As shown in Table 1, the majority of 327 nurse managers were female (99.39%) and married (95.41%), and 67.89% of participants were over 41 years old. Most of them held a bachelor’s degree (78.59%) and gained the professional title of nurse-in-charge and above (96.54%). Approximately 67.90% of participants had worked as nurse managers for more than six years. Additionally, 30.27% of nurse managers exercised less than once a month and most participants (92.05%) kept a 24-h mobile phone on.

**Table 1:** Characteristics of nurse managers and univariate analysis of sleep disorders with different demographic characteristics (*n* = 327)

Characteristics	*n*	%	PSQI score (Mean ± SD)	*t/F*	*p* value
Gender				0.247	0.805^a^
Male	2	0.6	7.00 ± 2.83		
Female	325	99.4	6.48 ± 2.96		
Age (years)				3.946	0.020^b^
≤ 30	11	3.4	4.55 ± 3.33		
31–40	94	28.7	6.12 ± 2.40		
≥ 41	222	67.9	6.73 ± 3.11		
Marital status				2.377	0.094^b^
Single	9	2.8	7.78 ± 2.77		
Married	312	95.4	6.40 ± 2.91		
Widowed/divorced	6	1.8	8.50 ± 4.76		
Education level				0.092	0.964^b^
High school/ technical school	2	0.6	7.00 ± 2.83		
Junior college and below	57	17.4	6.56 ± 3.13		
Bachelor's degree	257	78.6	6.44 ± 2.96		
Master's degree and above	11	3.4	6.82 ± 2.27		
Professional title				0.449	0.773^b^
Nurse	3	0.9	4.67 ± 3.21		
Nurse practitioner	41	12.5	6.59 ± 2.59		
Nurse-in-charge	203	62.1	6.54 ± 2.99		
Associate chief nurse	76	23.3	6.30 ± 3.08		
Chief nurse	4	1.2	7.25 ± 2.88		
Seniority in a nurse manager position (years)				0.587	0.672^b^
< 3	49	15.0	6.57 ± 2.81		
3–5	56	17.1	6.36 ± 2.83		
6–10	101	30.9	6.46 ± 2.77		
11–20	108	33.0	6.67 ± 3.17		
≥ 21	13	4.0	5.39 ± 3.82		
Frequency of exercise (times)				5.888	0.001^b^
Never	38	11.6	8.00 ± 3.62		
Rarely (≥ 1/quarter)	61	18.7	7.03 ± 3.02		
Occasionally (≥ 1/month)	107	32.7	6.25 ± 2.59		
Frequently (≥ 1/week)	121	37.0	5.93 ± 2.83		
Keeping a 24-h mobile phone on				-2.193	0.029^a^
Yes	301	92.0	6.59 ± 2.91		
No	26	8.0	5.23 ± 3.35		

*Abbreviation*: *PSQI* Pittsburgh Sleep Quality Index, *SD* standard deviation

^a^p-value of independent samples t test

^b^p-value of one-way analysis of variance (ANOVA)

### Sleep quality and coping style of respondents

The average PSQI score of nurse managers was 6.48 ± 2.96 and the prevalence of sleep disorders among participants was 43.7%. Each dimension’s mean score of sleep quality was presented in Table 2, with much lower scores for sleep efficiency (0.34 ± 0.68) and sleep medication (0.11 ± 0.48) than other dimensions. And the average scores for active coping and passive coping dimensions were 25.59 ± 5.53 and 8.21 ± 3.54 respectively (Table 2).

**Table 2:** Scores on sleep quality and coping style (*n* = 327)

Scales and Dimensions	Minimum	Maximum	Mean	*SD*
PSQI	1.00	17.00	6.48	2.96
Subjective sleep quality	0	3.00	1.06	0.79
Sleep latency	0	3.00	1.33	0.84
Sleep duration	0	3.00	1.28	0.74
Sleep efficiency	0	3.00	0.34	0.68
Sleep disturbance	0	3.00	1.07	0.55
Sleep medication	0	3.00	0.11	0.48
Daytime dysfunction	0	3.00	1.28	1.02
SCSQ				
Active coping	12.00	36.00	25.59	5.53
Passive coping	0	18.00	8.21	3.54

*Abbreviations*: *PSQI* Pittsburgh Sleep Quality Index, *SCSQ* Simplified Coping Style Questionnaire; *SD* standard deviation

### Correlations between sleep quality and demographic characteristics and coping style

According to the univariate analysis (Table 1), there were statistically significant differences in sleep quality among nurse managers of different age groups (*F* = 3.946, *p* = 0.020), frequency of exercise (*F* = 5.888, *p* = 0.001) and whether to keep a 24-h mobile phone on (*t* = 2.193, *p* = 0.029). In Table 3, Pearson’s correlation analysis showed that PSQI score was highly associated with active coping style (*r* = -0.599, *p* < 0.01) and passive coping style (*r* = 0.575, *p* < 0.01).

**Table 3:** Relationship between sleep quality and coping style (*n* = 327, *r*)

Variables	Active coping	Passive coping
Total score of PSQI	-0.599^**^	0.575^**^
Subjective sleep quality	-0.488^**^	0.358^**^
Sleep latency	-0.354^**^	0.309^**^
Sleep duration	-0.140^*^	0.241^**^
Sleep efficiency	-0.216^**^	0.300^**^
Sleep disturbance	-0.454^**^	0.396^**^
Sleep medication	-0.232^**^	0.278^**^
Daytime dysfunction	-0.471^**^	0.421^**^

*Abbreviation*: *PSQI* Pittsburgh Sleep Quality Index

^*^p < .05 (double-tailed), ^**^p < .01 (double-tailed)

### Influencing factors of sleep quality

The variables showing statistically significant association with a t test, ANOVA or correlation analysis were selected as the independent variables, and PSQI score was selected as the dependent variable to construct a multiple linear regression equation. As shown in Table 4, the result showed that active coping style (*β* = -0.418, *p* < 0.001), passive coping style (*β* = 0.393, *p* < 0.001), age (≥ 41 years old; *β* = 0.116, *p* = *0.003*) and exercise frequently (*β* = -0.135, *p* = 0.001) were significant predictors of poor sleep quality, collectively explaining 52.0% of the total variance of sleep quality (*F* = 89.465, *p* < 0.001).

**Table 4:** Multiple linear regression analysis of the influencing factors of sleep quality

Variables	Unstandardized Coefficients		Standardized	*t*	*p* value
	**B**	**SE**	**Coefficients (Beta)**		
Constant	9.322	0.753		12.381	< 0.001
Active coping	- 0.224	0.023	- 0.418	- 9.944	< 0.001
Passive coping	0.328	0.035	0.393	9.449	< 0.001
Age (≥ 41) (Reference: < 30)	0.732	0.245	0.116	2.987	0.003
Frequency of exercise: Frequently (Reference: Never)	- 0.827	0.242	- 0.135	- 3.412	0.001

*R* = 0.726, *R*^*2*^ = 0.526, adjusted *R*^*2*^ = 0.520, *F* = 89.465, *p* < .001

## Discussion

As far as we know, this study is the first to investigate the sleep quality of Chinese nurse managers during COVID-19 regular prevention and control and identify its influencing factors. Coping style, age and frequency of exercise were found as significant predictors of sleep quality among nurse managers.

In this study, 43.7% of participants suffered from sleep disorders. This finding was consistent with the result of 42.9% reported by Yang et al. (2012) [11], but higher than the result of 30% reported by Sun et al. (2019) [13] during a non-epidemic period in China. It is possibly due to higher role stress and duties of nurse managers in the context of COVID-19 regular prevention and control. During the COVID-19 pandemic, sleep disorders among frontline nurses have been widely reported in studies. For example, a study of 100 frontline nurses fighting against COVID-19 in Wuhan found that 60% of participants had sleep disorders, which was higher than the prevalence of nurse managers’ in this study [30]. This is partly because the study mentioned above was conducted in February 2020 when the COVID-19 pandemic was at its peak in China and there was a shortage of nurses. Conscripted frontline nurses were under huge workloads and prolonged working hours and were at risk of being infected. Under such circumstances, nurses were more vulnerable to sleep disorders. But it should not be ignored that there still was a fairly high incidence rate of sleep disorders of nurse managers in our study, which is expected to persist in the future. Our result highlights the importance and necessity of recognizing and putting efforts to solve their sleep problems. Besides, the score of each dimension of PSQI for nurse managers differed greatly, with much lower means for sleep medication and sleep efficiency dimensions than the others, which was in line with the result of Jahrami et al. (2021)’s study on medical staff [31]. Nurse managers, acting as professional healthcare workers, were proficient in the drug side effects and dependence, which may explain their less use of sleep pills. For sleep efficiency, which was defined as the ratio of total sleep time to time in bed and can be affected by various factors, such as physical and mental health and cultural or environmental factors [32]. Further studies are required to investigate the causes affecting the sleep efficiency of nurse managers.

We revealed that coping style was an important influencing factor of sleep quality among nurse managers. Specifically, active coping was adversely associated with poor sleep quality, whereas negative coping was positively related to it. Similar results were also found in previous studies [17, 33]. The underlying mechanism might be that passive coping leads to the decline of mood states and further the sleep disorders [34]. Sadeh et al. (2004) also thought that individuals with a passive response to stress tend to reduce emotional regulation and increase negative appraisal of stress, leading to hyper-arousal at bedtime that is associated with compromised sleep [35]. It follows that further studies could be conducted to investigate the interactions among stress, coping style, sleep quality and mental health of nurse managers. And healthcare administrators could provide nurse managers with professional psychological assistance like implementing the positive reappraisal coping intervention to promote their emotional regulation when experiencing stress [36], so as to promote nurse managers’ sleep quality.

As expected, our study also showed that nurse managers with older age (≥ 41 years old) were more susceptible to sleep disorders. A survey including 2007 general population in Sao Paulo found similar evidence that individuals aged 35 or above seemed to experience a higher level of sleep disorders [37]. Among nurses, older age was also found to be a risk factor for sleep disorders [9]. Generally, sleep problems have already been the well-established normal alterations in sleep physiology with age [38]. Especially for older (≥ 41 years old) female nurse managers, most of whom were either in menopausal transition or menopausal when rapid changes in estrogen levels, vasomotor instability and psychological symptoms were observed [39]. And it has been reported that these above changes can lead to the prevalence of women’s insomnia of 51–77% [39]. Hence, the mechanism of neurobiological changes may explain poor sleep quality of older nurse managers. Compared to their younger counterparts, middle-aged and old nurse managers have more family responsibilities and financial burdens and are also more prone to suffer from negative life events, such as divorces and chronic physical diseases [9, 40]. In this light, older nurse managers may experience worse sleep quality. Therefore, hospital administrators could provide older nurse managers more break time with fewer times on-call, equip them with assistants to take some responsibilities if possible, and reduce their workloads through optimizing work processes to alleviate age-related sleep disorders.

Finally, our result indicated that a higher frequency of exercise (≥ 1/week) had a positive impact on nurse managers’ sleep quality, which was consistent with the results of previous studies focusing on nurse managers [13] and nurses [41]. In addition, two meta-analyses also concluded that exercise could significantly reduce sleep latency and medication use of adults aged over 40 and improve sleep quality of middle-aged women [42, 43]. This is possibly because increased energy consumption, endorphin secretion and body temperature brought by exercise facilitate nurse managers’ sleep for recuperation of their bodies [42, 43]. And another study suggested that exercise as a means of active coping could potentially reduce stress levels and therefore has a positive effect on sleep quality [20]. Therefore, appropriate exercise training should be encouraged in nurse managers.

This study has several limitations. Firstly, the causal relationships between identified influencing factors and sleep quality cannot be drawn due to a cross-sectional design. Further longitudinal research is thus required to verify our findings. Secondly, the COVID-19 pandemic is continuing and changing, and studies investigating nurse managers’ sleep quality over longer periods are now needed. Thirdly, other factors that were not included in this study could also potentially affect sleep quality of nurse managers, such as bed capacity and occupancy rate in the ward, the number of staff they are managing, and the distance from the workplace to the accommodation. Therefore, further studies assessing the associations between sleep quality and these potential factors could provide additional information. Lastly, we conducted this study in 14 hospitals, but they were both located in Xiangyang City and the generalization of study results may be limited. Thus, research with a multi-regional sample will be required in the future.

## Conclusions

This study indicated that there was a fairly high percentage of nurse managers suffered from sleep disorders during regular prevention and control of the COVID-19, which may become chronic and long-lasting in the future and should be paid enough attention. Coping style, age and frequency of exercise were significant influencing factors of sleep quality in this study. In view of this, nurse managers should be encouraged to adopt active coping strategies against stress and take appropriate exercise. In addition, hospital superior administrators should provide nurse managers with professional psychological assistance facilitating coping and create reasonable human resources allocation and role configuration to improve their sleep quality and well-being, thereby contributing to desirable management and quality patient care.

## Data Availability

The datasets generated and/or analyzed during the current study are not publicly available due to agreements with participants who restricted data sharing but are available from the corresponding author on reasonable request.

## Abbreviations

COVID-19: Corona Virus Disease-2019
PSQI: Pittsburgh Sleep Quality Index
SCSQ: Simplified Coping Style Questionnaire

## References

[1] Kang L, Ma S, Chen M, Yang J, Wang Y, Li R (2020). Impact on mental health and perceptions of psychological care among medical and nursing staff in Wuhan during the 2019 novel coronavirus disease outbreak: a cross-sectional study. Brain Behav Immun.

[2] Gab Allah AR (2021). Challenges facing nurse managers during and beyond COVID-19 pandemic in relation to perceived organizational support. Nurs Forum.

[3] Jiang Z, Wang S, Shen Z, Zhao X, Wang F, Chen Y (2022). Nurses' experience of work stress related to COVID-19 regular prevention and control in China: a qualitative study. J Nurs Manag.

[4] Zheng Y, Xiao L, Xie Y, Wang H, Wang G (2020). Prevalence and characteristics of obsessive-compulsive disorder among urban residents in Wuhan during the stage of regular control of coronavirus disease-19 epidemic. Front Psychiatry.

[5] Jackson J, Nowell L (2021). 'The office of disaster management' nurse managers' experiences during COVID-19: a qualitative interview study using thematic analysis. J Nurs Manag.

[6] Arakelian E, Rudolfsson G (2021). Managerial challenges faced by Swedish nurse managers in perioperative settings- a qualitative study. BMC Nurs.

[7] Shirey MR, Ebright PR, McDaniel AM (2008). Sleepless in America: nurse managers cope with stress and complexity. J Nurs Adm.

[8] Giorgi F, Mattei A, Notarnicola I, Petrucci C, Lancia L (2018). Can sleep quality and burnout affect the job performance of shift-work nurses? A hospital cross-sectional study. J Adv Nurs.

[9] Zhou Y, Yang Y, Shi T, Song Y, Zhou Y, Zhang Z (2020). Prevalence and demographic correlates of poor sleep quality among frontline health professionals in Liaoning Province, China During the COVID-19 outbreak. Front Psychiatry.

[10] Sigursteinsdóttir H, Skúladóttir H, Agnarsdóttir T, Halldórsdóttir S (2020). Stressful factors in the working environment, lack of adequate sleep, and musculoskeletal pain among nursing unit managers. Int J Environ Res Public Health.

[11] Yang YX, Li JP (2012). Investigation on sleep quality of head nurses in Sichuan Province. Chin J Mod Nurs.

[12] Steege LM, Pinekenstein BJ, Arsenault Knudsen É, Rainbow JG (2017). Exploring nurse leader fatigue: a mixed methods study. J Nurs Manag.

[13] Sun X, Li J, Wang Y, Wang GH (2019). Sleep quality and mental health of head nurses in tertiary hospitals. Chin Pract Prev Med.

[14] White JH (2021). A phenomenological study of nurse managers' and assistant nurse managers' experiences during the COVID-19 pandemic in the United States. J Nurs Manag.

[15] Kim-Godwin YS, Lee MH, Logan JG, Liu X (2021). Factors influencing sleep quality among female staff nurses during the early COVID-19 pandemic in the United States. Int J Environ Res Public Health.

[16] Zhan Y, Liu Y, Liu H, Li M, Shen Y, Gui L (2020). Factors associated with insomnia among Chinese front-line nurses fighting against COVID-19 in Wuhan: a cross-sectional survey. J Nurs Manag.

[17] Li Y, Cong X, Chen S, Li Y (2021). Relationships of coping styles and psychological distress among patients with insomnia disorder. BMC Psychiatry.

[18] Yoshida K, Otaka H, Murakami H, Nakayama H, Murabayashi M, Mizushiri S (2018). Association between insomnia and coping style in Japanese patients with type 2 diabetes mellitus. Neuropsychiatr Dis Treat.

[19] Sun GW, Yang YL, Yang XB, Wang YY, Cui XJ, Liu Y (2020). Preoperative insomnia and its association with psychological factors, pain and anxiety in Chinese colorectal cancer patients. Support Care Cancer.

[20] Otsuka Y, Kaneita Y, Itani O, Nakagome S, Jike M, Ohida T (2017). Relationship between stress coping and sleep disorders among the general Japanese population: a nationwide representative survey. Sleep Med.

[21] Li X, Guan L, Chang H, Zhang B (2014). Core self-evaluation and burnout among Nurses: the mediating role of coping styles. PLoS ONE.

[22] Guo J, Feng XL, Wang XH, van IMH. Coping with COVID-19: Exposure to COVID-19 and negative impact on livelihood predict elevated mental health problems in Chinese adults. Int J Environ Res Public Health. 2020;17(11):3857. 10.3390/ijerph17113857. 10.3390/ijerph17113857 PMC7312167 32485859

[23] Johnston KM, Lakzadeh P, Donato BMK, Szabo SM (2019). Methods of sample size calculation in descriptive retrospective burden of illness studies. BMC Med Res Methodol.

[24] Buysse DJ, Reynolds CF, Monk TH, Berman SR, Kupfer DJ (1989). The Pittsburgh Sleep Quality Index: a new instrument for psychiatric practice and research. Psychiatry Res.

[25] Liu XC, Tang MQ, Hu L, Wang AZ, Wu HX, Zhao GF (1996). Reliability and validity of Pittsburgh sleep quality index. Chin J Psychiatry.

[26] Kong F, Li H, Xu G, Ying Y, Gong Q, Zhao J (2018). Association of dietary behaviors and sleep quality: results from the adults chronic diseases and risk factors survey of 2015 in Ningbo, China. Int J Environ Res Public Health.

[27] Xiong W, Liu H, Gong P, Wang Q, Ren Z, He M (2019). Relationships of coping styles and sleep quality with anxiety symptoms among Chinese adolescents: a cross-sectional study. J Affect Disord.

[28] Folkman S, Lazarus RS (1985). If it changes it must be a process: study of emotion and coping during three stages of a college examination. J Pers Soc Psychol.

[29] Xie YN (1998). Reliablility and validity of the Chinese version of simplified coping style questionaire. Chin J Clin Psychol.

[30] Tu ZH, He JW, Zhou N (2020). Sleep quality and mood symptoms in conscripted frontline nurse in Wuhan, China during COVID-19 outbreak: a cross-sectional study. Medicine (Baltimore).

[31] Jahrami H, BaHammam AS, AlGahtani H, Ebrahim A, Faris M, AlEid K (2021). The examination of sleep quality for frontline healthcare workers during the outbreak of COVID-19. Sleep Breath.

[32] Park E, Lee HY, Park CS (2018). Association between sleep quality and nurse productivity among Korean clinical nurses. J Nurs Manag.

[33] Ren Z, Zhang X, Shen Y, Li X, He M, Shi H (2021). Associations of negative life events and coping styles with sleep quality among Chinese adolescents: a cross-sectional study. Environ Health Prev Med.

[34] Hoyt MA, Thomas KS, Epstein DR, Dirksen SR (2009). Coping style and sleep quality in men with cancer. Ann Behav Med.

[35] Sadeh A, Keinan G, Daon K (2004). Effects of stress on sleep: the moderating role of coping style. Health Psychol.

[36] Nowlan JS, Wuthrich VM, Rapee RM (2016). The impact of positive reappraisal on positive (and negative) emotion among older adults. Int Psychogeriatr.

[37] Maluly M, Dal Fabbro C, Andersen ML, Herrero Babiloni A, Lavigne GJ, Tufik S (2020). Sleep bruxism and its associations with insomnia and OSA in the general population of Sao Paulo. Sleep Med.

[38] Waller KL, Mortensen EL, Avlund K, Osler M, Fagerlund B, Lauritzen M (2016). Subjective sleep quality and daytime sleepiness in late midlife and their association with age-related changes in cognition. Sleep Med.

[39] Dzaja A, Arber S, Hislop J, Kerkhofs M, Kopp C, Pollmächer T (2005). Women's sleep in health and disease. J Psychiatr Res.

[40] Nagle C, Omonaiye O, Bennett PN (2021). Valuing nurse and midwifery unit managers' voices: a qualitative approach. BMC Nurs.

[41] Dong H, Zhang Q, Zhu C, Lv Q (2020). Sleep quality of nurses in the emergency department of public hospitals in China and its influencing factors: a cross-sectional study. Health Qual Life Outcomes.

[42] Rubio-Arias J, Marín-Cascales E, Ramos-Campo DJ, Hernandez AV, Pérez-López FR (2017). Effect of exercise on sleep quality and insomnia in middle-aged women: a systematic review and meta-analysis of randomized controlled trials. Maturitas.

[43] Yang PY, Ho KH, Chen HC, Chien MY (2012). Exercise training improves sleep quality in middle-aged and older adults with sleep problems: a systematic review. J Physiother.

